# Novel Biocompatible and Biodegradable PCL-PLA/ Iron Oxide NPs Marker Clip Composite for Breast Cancer Biopsy

**DOI:** 10.3390/polym10121307

**Published:** 2018-11-26

**Authors:** Angel R. Hernandez-Martinez, Gustavo A. Molina, Rodrigo Esparza, Ángel Luis Rodríguez, Martha Cruz-Soto, Eloy Rodríguez-de León, Domingo Rangel, Miriam Estévez

**Affiliations:** 1 Centro de Física Aplicada y Tecnología Avanzada (CFATA), Universidad Nacional Autónoma de México (UNAM), Blvd. Juriquilla 3000, 76230 Querétaro, Mexico; resparza@fata.unam.mx (R.E.); alrodriguez@fata.unam.mx (Á.L.R.); ranged@fata.unam.mx (D.R.); 2 Posgrado en Ciencia e Ingeniería de Materiales, Centro de Física Aplicada y Tecnología Avanzada (CFATA), Universidad Nacional Autónoma de México (UNAM), Blvd. Juriquilla 3000, 76230 Querétaro, Mexico; gustavomolina21@gmail.com; 3 Universidad del Valle de México, Campus Querétaro, Blvd. Juriquilla 3000, Juriquilla, 76230 Querétaro, Mexico; martha.cruzso@uvmnet.edu; 4 Posgrado en Ciencias Químico Biológicas, Faculty of Chemistry, Autonomous University of Querétaro, Cerro de las Campanas, 76010 Querétaro, Mexico; eloy.q22@gmail.com

**Keywords:** marker clip, PLA-PCL, metallic nanoparticles, breast cancer, composite, biopsy

## Abstract

Strength and biocompatibility of composite materials (using a polymer matrix) are used in medicine for various devices such as prostheses and marker clips (biomarkers). Marker clips indicate the site of a lesion in the body, specifically for breast cancer diagnosis or treatment. In general, marker clips are made of steel or titanium, but lately, materials containing biodegradable polymers had been proposed. Our hypothesis is that a copolymer of polylactic acid and poly(ε-caprolactone) (PLA-PCL) could be used as marker clip material. After evaluating different polymer rates performance, metallic nanoparticles (NPs) were included to enhance the stability of the best copolymer and a marker clip prototype was proposed. Characterization of nanoparticles was made by dynamic light scattering (DLS), X-ray diffraction (XRD) and magnetic measurements. Mechanical, thermal and radiopacity properties were evaluated for composites formulation. In vitro, radiopaque experiments showed that BM-2 composite had the best performance. In vivo experiments showed that, after five months, the marker clip prototype maintained its shape, visibility and contrast properties. In consequence, a novel formulation of composite (PLA-PCL/metallic nanoparticles) is suitable for further studies as an alternative material for marker clips for breast cancer lesions.

## 1. Introduction

Materials engineering contribute to improving medical options in current treatments. The composites are one example of it, which engineered materials with two or more components that offer different physical properties and could be combined synergistically [1,2]. The strength and biocompatibility of composite materials (using a polymer matrix) are used in orthopedics and constructing prostheses [1]. Other composites are used as marker clips (biomarkers), which are devices that mark the site of a lesion in the body, specifically for breast cancer diagnosis/treatment. Before marker clips existence, medical personnel detected lesions after percutaneous breast biopsies only through ultrasound, which was less precise [3]. Marker clips are currently helping the radiologist to deal with multiple lesions and the surgeon to mark the margins of extensive disease or guide intraoperative tumor resection. Marker clips are safe in general; some exceptional cases of women presented an allergic reaction to the metallic clip [3,4]. Commercial breast markers are expensive; therefore, some techniques had been developed to decrease costs, such as the Montreal technique, that accomplished two commercially available titanium clips as low-cost systems [3].

Manufacturing materials of these devices must have monodispersity, biocompatibility, and flexible design [5,6]. The material biocompatibility indicates its ability to perform in conjunction with a living system [1]; therefore, marker clips must be biocompatible and minimize the risk of infections. Materials used for marker clips could be metals or metals surrounded by filling material. Bioresorbable collagen plug of bovine origin, pellets of poly(lactic acid) (PLA), poly(glycolic acid) (PGA), and poly(vinyl alcohol) (PVA), as well as poly(ethylene glycol) hydrogels, had been used as filling materials for packing stainless steel or titanium [3,7]. PLA and PGA are biodegradable polymers that play an important role in the medicine area; particular applications use engineered materials of these polymers with adjustments on their physical and mechanical properties. Polymers degrade by hydrolysis and enzymatic activity depending on several factors such as molecular structure, crystallinity, water diffusion rate into the polymer, and the stereoisomeric content [8,9]. PLA, PGA, and their derivatives have originated a large family of materials with a wide range of engineered properties and biological responses. Among them, a variety of copolymers had been synthesized.

PLA is a bioabsorbable polymer, widely used because its monomers could be produced from a non-toxic raw material; its polymeric unit is the lactic acid (2-hydroxypropanoic acid), that being a chiral molecule has two enantiomers, l- and d-lactic acid [9]. PLA has stereoisomers, such as poly (l-lactide) (PLLA), poly(d-lactide) (PDLA), and poly (dl-lactide) (PDLLA) [9,10]. Lactic acid could be obtained by fermentation of sugars from cornstarch and sugarcane. PLA could be synthesized by polycondensation, azeotropic dehydration, ring opening polymerization and enzymatic polymerization [9]. Other polymers had been accepted to use them as medical devices, such as poly(dioxanone), poly(trimethylene carbonate) copolymers, and poly(ε-caprolactone) (PCL) homopolymers and copolymers. Additionally, poly(anhydrides) and poly(orthoesters) had been studied as alternative medical materials [11]. PCL is a semi-crystalline biodegradable linear aliphatic polyester susceptible, it presents slow degradation and it is also biocompatible [12]. Considering the aforementioned biocompatible materials, our hypothesis is that a copolymer of PLA-PCL could be used as marker clip material. At room temperature, PCL has low tensile modulus and high elongation at break while PLA shows high modulus and low elongation at break due to it is higher glass transition temperature [12]. Therefore, a copolymer could have better performance as a medical device.

As we are using a copolymer, other factors could have an impact on marker clip degradation, e.g., the copolymer ratio, therefore we decide to include iron oxide nanoparticles (IONPs) in the formulation to enhance stability of the copolymer and also because they are known to be biological tolerated, benign, and their toxicity, metabolism, and pharmacokinetics have been well studied [13,14]. Yu et al. investigated toxicity of iron nanoparticles in cells using 5 and 30 nm in porcine aortic endothelial cells. They found that cytotoxicity depends on nanoparticles size, the production of reactive oxygen species (which can lead to cell death) increased with the nanoparticle size [15]. Then, IONPs in the marker clip formulation should have a size less than 30 nm and are noteworthy to mention that several IONPs have been approved for clinical applications [14,16,17].

On the other hand, it has been suggested that marker clips should not have a biological shape (for easy identification) and an adequate size to fit into a 12G biopsy syringe (∅in = 2.7 mm) [15]. Their shape could be a ribbon, a ribbon wing coil, a “T” cylinder, a breast cancer awareness symbol, a coil, or “S”, “X”, “O”, “M” [3,18]. We proposed a prototype with a novel design of the biomarker shape. These novel markers would avoid having a second surgery for removing the non-absorbable metal clip, leading to reduced stress and additional pain to the patient, as well as a reduction in total costs.

## 2. Materials and Methods

### 2.1. Ethical Approval of Animals Used for This Study

Procedures involving animals were revised and approved by the Ethics Committee at “Universidad del Valle de Mexico”, campus Queretaro in compliance with current regulations of scientific content in research using animals. In other words, applicable international and national guidelines for care and use of animals were followed; procedures performed in our studies were conducted in accordance with the ethical standards of our institution. Approval of the Ethics Committee with reference number CSUVMI 2016-002 was added as supplementary material.

### 2.2. Materials

Lactic acid solution (85%), 2-ethylhexanoic acid tin (II) salt (~95%), ferric chloride hexahydrate (FeCl_3_·6H_2_O, 97%), ferrous chloride (FeCl_2_, 98%), poly(*ε*-caprolactone) (average molecular weight; Mw ~14,000) were purchased from Sigma-Aldrich (Toluca, Mexico). Glycerol anhydrous (99.8%), ammonium hydroxide (NH_4_OH, ~29%), sodium citrate tribasic dihydrate (Na_3_C_6_H_5_O_7_·2H_2_O, ~98%) and ethanol (EtOH, >99.5%) were purchased from J.T Baker (CDMX, Mexico). Experiments were conducted using deionized water (de-H_2_O) and chemicals were used as received without further purification.

### 2.3. Polymerization of Poly(Lactic Acid)

The polylactic acid was obtained through a polycondensation reaction [19]. First, 1 mol of the lactic acid aqueous solution was mixed with 1 mL of sulfuric acid (azeotropic distillation) by magnetically stirring, and then refluxed at 120 °C/atmospheric pressure for 3 h. Then, 0.0005 mol of 2-ethylhexanoic acid tin were added and mixed. Solvents were evaporated under vacuum at 60 °C/45 mbar for 12 h. Finally, the dehydrated product was recovered and stored in vials until further studies.

### 2.4. Synthesis of Iron Oxide Nanoparticles (IONPs)

IONPs were synthesized by mixing 1 M FeCl_3_ and FeCl_2_ aqueous solutions (molar ratio 1:1) with a sodium citrate solution containing 25% of citrate ions (regarding iron ions) and 1 M NH_4_OH solution until the pH reached 9. The solution was kept under magnetically stirring at 70 °C during 2 h and maintained in an inert atmosphere by constant bubbling of N_2_. The black precipitate obtained was collected using a ~12,100 gauss neodymium magnet and subjected to a three-cycle washing process using de-H_2_O and EtOH, and finally dried under vacuum.

### 2.5. Copolymer/Composite Formulations

PCL was heated until it melted (~40 °C), then PLA was slowly poured until a homogeneous dissolution was produced. Then, the blend was injected into a cast and frozen for 24 h. Based on the degradation rate experiments of each blend under physiological conditions (37 °C/pH 7.42), the most suitable blends were selected for combining them with IONPs. The formulations obtained, according to PCL/PLA/IONPs ratio, in grams were: 4.50/0.50/0.0 (labeled PB-1); 4.75/0.25/0.0 (PB-2); 4.90/0.10/0.0 (PB-3); 4.75/0.25/12.64 × 10^−3^ (BM-1); and 4.75/0.25/36.86 × 10^−3^ (BM-2); and degradation rate was evaluated for each one.

### 2.6. Marker Clip Prototype Fabrication

**Figure 1:**
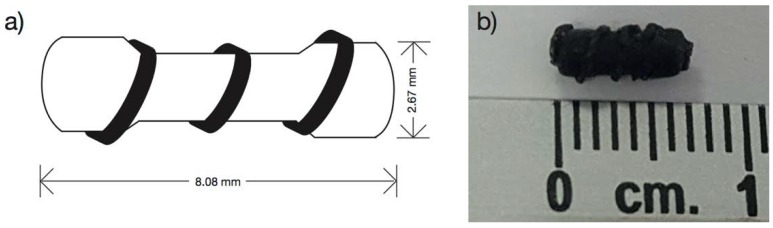
(**a**) Marker clip silicon cast design and (**b**) marker clip containing IONPs after 24 h of deep freeze.

A silicon cast was designed with the form of a 100 Ω commercial resistor enveloped with a copper wire of 24 AWG calibers (ø = 0.51 mm) (Figure 1a). The silicon cast developed is 2.67 mm wide and 8.08 mm long (Figure 1b). It was filled by solvent casting technique using formulations PB-1, PB-2, PB-3, BM-1, and BM-2 to produce the marker clip prototype.

### 2.7. Characterization

#### 2.7.1. Characterization of the IONPs

The IONPs characterization was based on particle size distribution by dynamic light scattering (DLS) and physical state by X-ray diffraction (XRD), transmission electron microscopy (TEM) and magnetic hysteresis.

For DLS, IONPs were dispersed in toluene for 10 min using an ultrasonic bath and three different measurements of each batch were made. These measures were carried outusing a Zetasizer Nano-S (Malvern Instruments, Malvern, UK) at 25 °C with a standard laser irradiation of 633 nm and the scattering was detected at 173°.

For XRD, samples were placed on an aluminum plate and analyzed by a RIGAKU Ultima-IV (Rigaku Americas Corp., Woodlands, TX, USA) with the following conditions, 2θ range from 5° to 80° steps of 0.022 θ and ~0.7 s per step, X-ray tube operated at 30 kV and 10 mA with CuKα (λ = 1.5406 Å) radiation.

For TEM analysis, JEOL-1230 TEM microscope (Tokyo, Japan) operating at an accelerating voltage of 100 keV was used. The samples were prepared via dropping diluted dispersions onto copper grids coated with carbon support film and subsequently drying at room temperature.

For magnetization curves, the sample was placed and glued on an acetate placeholder prior to measurements. The sample was analyzed by a MicroMag-2900 (Lake Shore Cryotronics Inc., Westerville, OH, USA), alternating magnetic field gradient magnetometer using a 10 kOe saturating field.

#### 2.7.2. Characterization of Marker Clip Formulations

The different copolymer/composite formulations characterizations were evaluated using a chemical structure analysis from Fourier transform infrared spectroscopy (FTIR), physical properties such as degradation rate, mechanical properties, thermal analysis by thermogravimetric analysis (TGA) and differential scanning calorimetry (DSC) and the IONPs distribution on the copolymer by electron dispersive X-Ray spectroscopy (EDS). Additionally, the characterization of the synthesized PLA (used for the copolymer/composite formulations) was conducted using proton nuclear magnetic resonance spectroscopy (^1^H NMR) on a Bruker Avance III HD 500 MHz instrument (Bruker Avance, Milton, ON, Canada), using CDCl3 as solvent and tetramethylsilane (TMS) as internal standard at 25 °C. Also gel permeation chromatography (GPC) was performed to determine PLA molecular weight on a Viscotek RImax chromatograph (Malvern Panalytical Ltd., Malvern, UK) using two PL mixed E columns with a Viscotek model 3580 refractive index detector and an injection volume of 100 µL of the samples through the autosampler. Chloroform was used as the mobile phase at a flow rate of 1 mL/min and molecular weight was calculated from a prepared polystyrene standard curve with a molar-mass dispersity close to 1.0. All measurements for GPC were made at 35 °C.

Degradation rate by hydrolysis of the different copolymers/composite formulation was evaluated by a gravimetric analysis under a physiological condition at 37 °C using a bicarbonate buffer solution (pH = 7.42).

In order to evaluate mechanical properties, a tensile testing equipment Zwick/Roell Z005 (Zwick/Roell Group, Ulm, Germany) was used to examine mechanical properties of the small cylinders (20 mm diameter, 4 mm length) obtained from all the PCL/PLA/IONPs different formulations, according to the Standard ASTM D 695-02. Compression rate was set to 1.0 mm/min and three different measurements were performed under 30% of maximum compression.

For thermal properties, thermogravimetric analysis (TGA) and thermal analysis by differential scanning calorimetry (DSC) of PLA, PCL, and PM-2 composite formulation; were conducted to determine their thermal degradation and thermal behavior respectively. A TGA/DSC Model 2 Stare System (Mettler-Toledo Intl. Inc., Columbus, OH, USA) was used. Samples of 5–12 mg were heated from 20 to 700 °C with a scan rate of 10 °C/min under a nitrogen atmosphere at a flow rate of 40 NmL/min (milliliter per minute at normal conditions of temperature and pressure).

The FTIR was evaluated on PLA, PCL, IONP´s, and PM-2 composite formulation by means of a Frontier MIR/NIR Spectrometer (Perkin Elmer, Waltham, MA, USA) under attenuated total reflectance (ATR) mode in the range from 400 to 4000 cm^−1^.

Finally, the distribution of IONPs on marker clip prototype was measured through EDS using a Bruker XFlash 6/60 Silicon Drift Detector coupled to a Hitachi SU8230 cold-field emission microscope (Hitachi America, Ltd., New York, NY, USA). Samples were placed on Al stubs without any conductor layer deposited and images were taken under secondary electron detector (SE) at 20 keV with a working distance of ~15 mm.

#### 2.7.3. Radiopacity Test of Marker Clip

The radiopaque properties of the composite markers formulated in this study were evaluated in both in vitro and in vivo phases. Images were acquired on the first day of implantation, three and four months after implantation using a dental imaging X-ray (COR-70/8-03, CORAMEX, Qro, Mexico). X-ray images were acquired to validate the radiopacity of the marker, to suggest a further stereotactic breast biopsy use. Also, images were taken using a digital mammography system (AMULET, FUJIFILM) with tube current = 21 mA, exposure time = 462 s, KVP = 24 kV, exhibition = 10 mAs, AGD = 0.14 mGy, a white/filter of tungsten/rhodium.

In vitro imaging was analyzed for two composite markers (BM-1 and BM-2) and one commercial metallic marker was implanted in a mammary gland model composed by adipose and muscle tissue and can be observed in the X-ray imaging (Figure 9). A custom MATLAB script (MathWorks Inc., Natick, MA, USA) was used to determine the radiopacity scale, on a grayscale of 8-bit, where the lowest value (black—0) was considered radiopaque and the highest value (white—255) was considered radiolucent.

**Figure 2:**
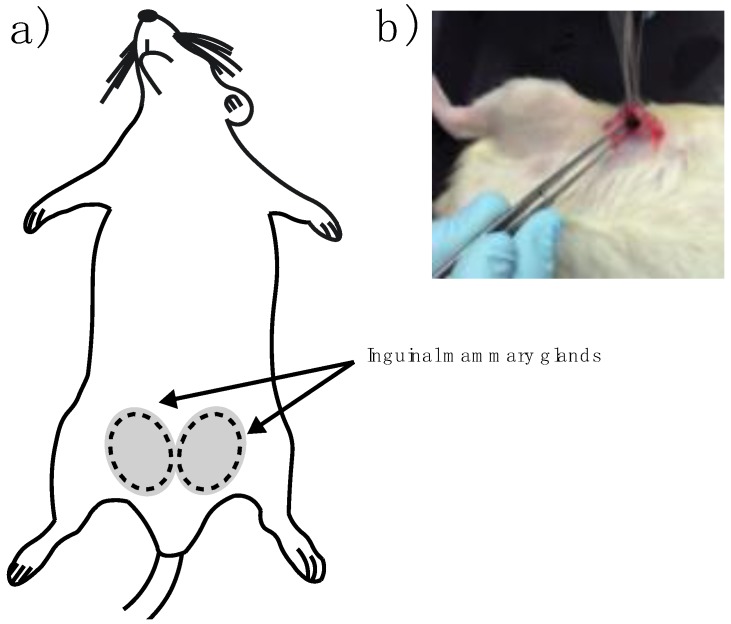
(**a**) Schematic representation of inguinal mammary glands of the rat and (**b**) composite marker clip implant into the inguinal mammary gland of Wistar rat by biopsy.

In vivo imaging experiments were performed on female Wistar rats (9–12 months old) inguinal mammary fat pads were used. Animals were cycling to ensure mammary fat pad architecture under the influence of sex steroids. One female rat served as a negative control with no implant and nine experimental adult rats were implanted. Briefly, rats were anesthetized by intraperitoneal injection with 1 mL per kg of 70/30 of ketamine/xylazine mixture. Then a fine incision was performed exposing the inguinal mammary fat pad to introduce the sterile BM-2 blend marker (Figure 2a,b). Two stitches were needed to keep the marker in place and a super oxidant solution was applied to avoid infections and to help healing of the wound.

## 3. Results

Animals used for this study were maintained in a cycle of 12 h light/12 h darkness with water and food ad libitum, under conditions of NOM-062-ZOO-1999 (Mexican Official Standard) concerning technical specifications for production, care and use of laboratory animals.

### 3.1. Characterization of the IONPs

**Figure 3:**
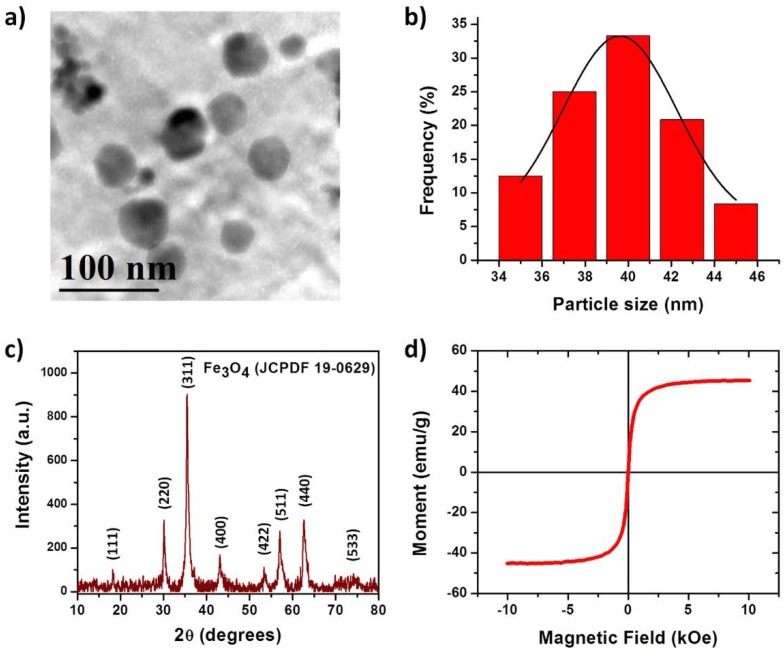
(**a**) TEM micrograph of iron oxide nanoparticles (IONPs) (Fe3O4 NPs), (**b**) particle size distribution determined by dynamic light scattering (DLS), (**c**) X-Ray diffraction pattern, and (**d**) hysteresis loop of IONPs at 298 K.

Low magnification TEM image of the IONPs sample is shown in Figure 3a. The morphology of the nanoparticles is hexagonal-like with faceted surface planes. Particle size distribution was determined using DLS technique, and results showed that small particles were obtained with a mean particle size of 39.6 ± 1.136 nm (Figure 3b), indicating that a homogeneous diameter was achieved with experimental conditions established in this work. X-Ray diffraction patterns of IONPs (Figure 3c) showed diffraction peaks of 2θ correspond to the (111), (220), (311), (400), (422), (511), (440), and (533) planes, which indicate the pure cubic phase of the Fe_3_O_4_ (JCPDF 19-0629). The average crystal size was calculated with the Debye–Scherrer equation [20] using the highest intensity XRD peak, namely (311), which predicted an average crystal size of 32.3 nm, that is in accordance with the size measured by DLS (considering that it measures the hydrodynamic diameter of the particles). A parameter used to define the size range of nanomaterials is called the “polydispersity index” (PDI) or “dispersity” (Đ) as recommended by IUPAC. The Đ is used to describe the degree of non-uniformity of a size distribution of particles and it is calculated from the DLS data or TEM images by the following equation; where σ is the standard deviation and “x bar” is the mean [21,22].

(1) $$Đ={\left(\frac{\sigma }{\bar{x}}\right)}^{2}$$

The dispersity of the IONPs from DLS data was of 8.2 × 10^−4^ and of 1.2 × 10^−3^ from TEM images, in both cases, the Đ values are lower than 0.05 thus the IONPs are highly monodisperse. The magnetic measurement on the IONPs was performed at 298 K. The coercivity and remnant magnetization curve obtained for the nanoparticles is presented in Figure 3d. As can be seen, the sample revealed typical superparamagnetic behavior given that the curve does not have any hysteresis loop, which is consistent with the superparamagnetic properties arising from the small size of the magnetic core [23]. The samples revealed mild remnant magnetization (Mr) and zero coercivity (Hc), the Mr value was estimated to be ≈45 emu/g, which is lower to the theoretically estimated for bulk Fe_3_O_4_ (92 emu/g).

### 3.2. Degradation Rate and Mechanical Properties

**Figure 4:**
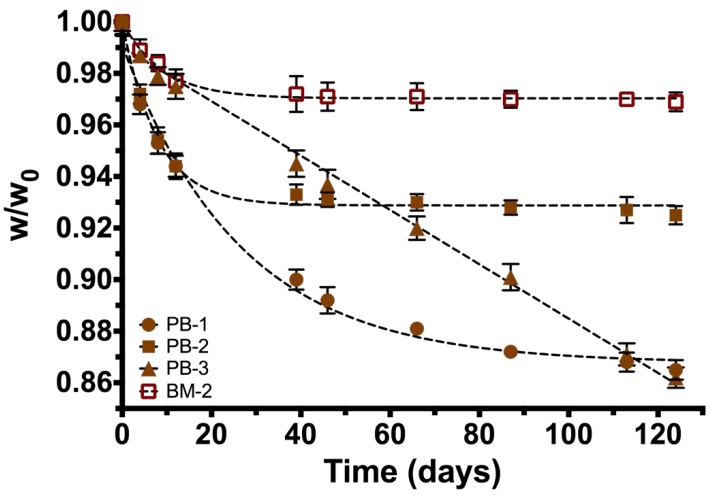
Degradation rates of copolymer formulations during hydrolysis process (days). PB-1, PB-2, PB-3: different copolymer formulations (poly(lactic acid) (PLA)/ poly(ε-caprolactone) (PCL) rates), BM-2—sample (PLA/PCL/ IONPs).

Figure 4 shows the degradation rate of the three initial polymer mixtures obtained in this work. The data was adjusted to describe an exponential decay model based on the response (weight loss) of the composite markers under physiological conditions:

(2) $$w=w_{0}\cdot e^{-\left(k\cdot t\right)}$$

where *w*_0_ is the weight loss at time *t* = 0 and *k* is the constant rate. Table 1 summarizes different constant rates and weight loss percentage for each of initial polymer solutions.

Also, it can be observed mechanical compression properties of the different formulations of copolymers on Table 2 built from the stress/strain curve of each sample from a linear fit on the elastic region. Compression analysis was performed because previous materials being used for a mammary gland biopsy, were an incremental stiffening along with the tissue, which is a characteristic of tumor progression [24,25], and the force interacting with the material should be compression and not tension.

### 3.3. Thermal Behavior of Composite

**Figure 5:**
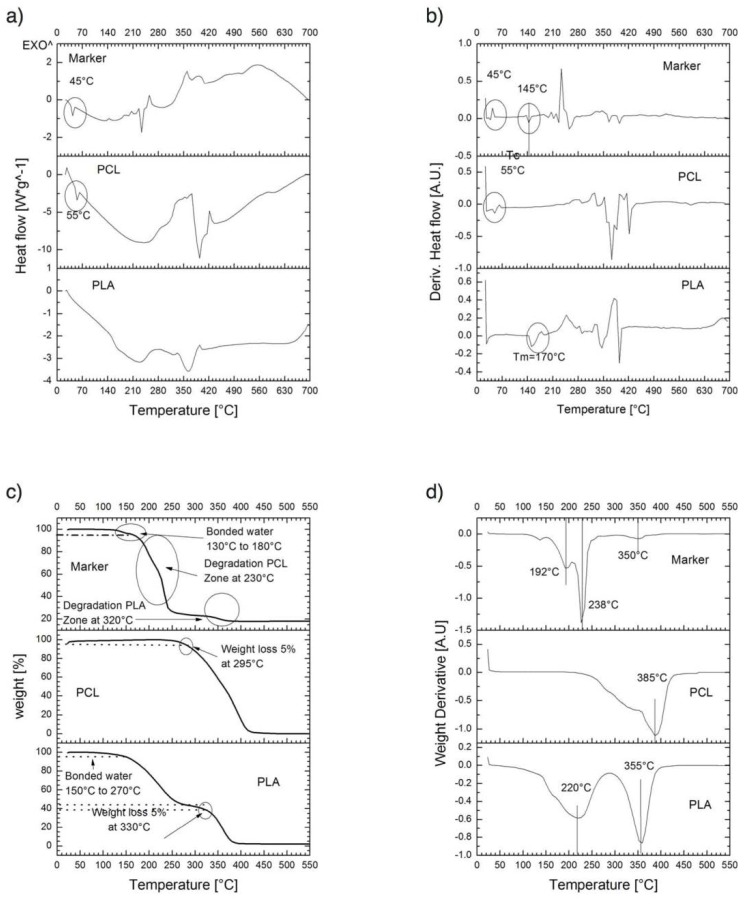
Thermal degradation properties for PLA, PCL, and composite marker: (**a**) thermogravimetric analysis (TGA), (**b**) thermogravimetric derivative (DTG) and thermal curves, (**c**) differential scanning calorimetry (DSC) heating curves and (**d**) first derivative curve of DSC curve.

Thermal behavior of formulations developed in this study is shown in Figure 5. A criterion of 5% weight loss (T5%) was determined to characterize initial thermal stabilities of formulations for thermogravimetric analyses. TGA thermogram for PLA showed a two-stage decomposition process; the first stage is related to the loss of physically bonded water below 270 °C, and the main decomposition step associated with PLA degradation occurs between 300–390 °C; also, ester groups hydrolysis was accelerated by groups (–COOH) and shown around 330 °C, this value is similar to previous reports for PLA with Mw from low to medium [26]. TGA thermogram of PCL showed a 5% weight loss at 295 °C. The thermogravimetric analyses of the composite marker showed three-stage decomposition process, the first stage between 130–180 °C related to the loss of physically bonded water, the second-stage was associated with degradation of PCL polymer chains due to rupture of polyester via pyrolysis reaction [27], and the third-stage was attributed to a combination of overlapping process between PCL degradation and PLA degradation, dominated by backbiting ester interchange reaction involving –OH chain ends [28].

In this figure, (Figure 5) it is also shown the DSC curves for PLA, PCL, and composite marker. DSC thermograms of PLA showed different endothermic transitions around 45, 160, and 359 °C that could be attributed to glass transition temperature (Tg), melting point, and the degradation temperature of the polymer obtained, respectively [29]. PCL thermograms exhibited different endothermic peaks, that have been related to moisture evaporation (55 °C) and structural decomposition (250 and 360 °C) of the polymer by other authors [30]. Composite showed DSC profiles with series of thermal transitions different from pure polymers.

**Figure 6:**
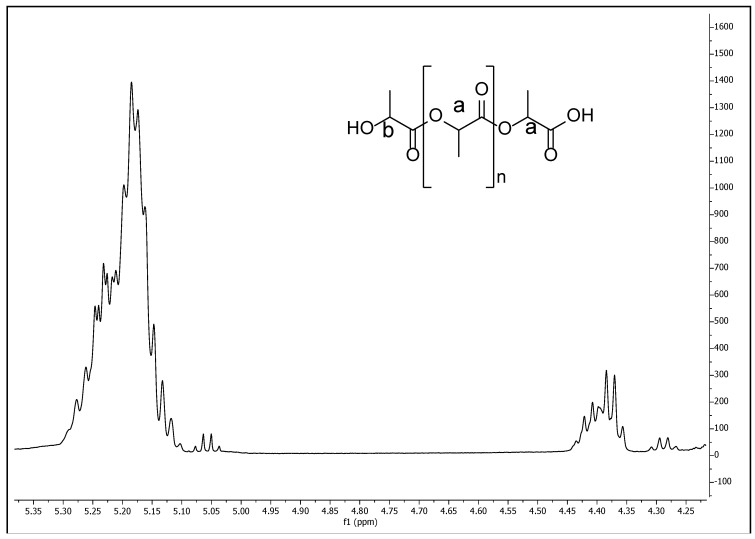
^1^H NMR (500 MHz) spectrum in CDCl_3_ for the poly(lactic acid) (*M_n_*(NMR)_aprox_ = 1440).

As mentioned previously; PLA has molecular weight from low to medium, which influences both the mechanical and the degradation properties. Thus, a ^1^H NMR was performed (Figure 6), there it can be observed a spectrum that corresponds to dl-lactic acid, with a methyne group at δ 5.20 (a) and a terminal methylene end group at δ 4.70 (b). A number average molecular weight (Mn) of 1.44 × 10^3^ g·mol^−1^ was obtained from the data of the spectrum. This was corroborated from the GPC characterization of the PLA, where values of 1.52 × 10^3^ and 1.97 × 10^3^ g·mol^−1^ were obtained for the Mn and for the weight average molecular weight (Mw), respectively. The polydispersity degree (PD) resulted 1.3.

### 3.4. Structural Analysis and IONPs Distribution on the Marker Clip

**Figure 7:**
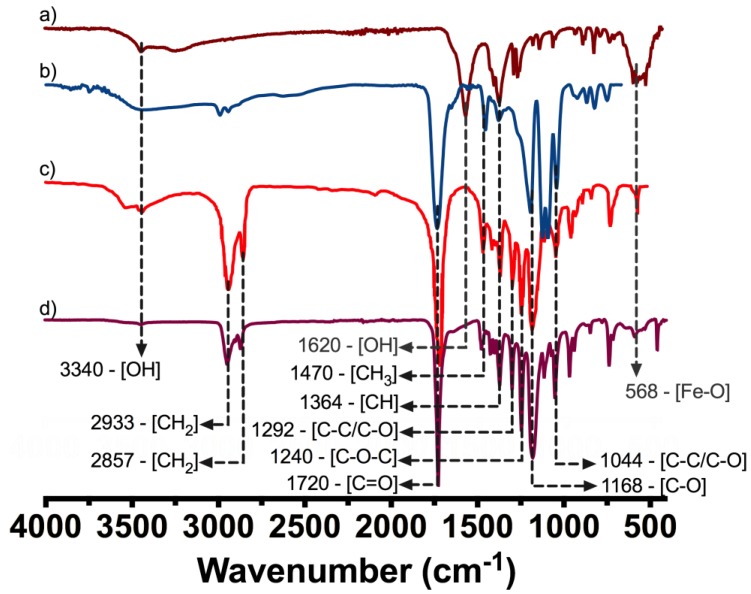
Fourier transform infrared spectroscopy (FTIR) spectrum from (**a**) IONPs, (**b**) PLA, (**c**) PLC and (**d**) BM-2 marker clip prototype (PLC/PLA/IONPs: 4.75/0.25/36.86·× 10^−3^).

The characteristic FTIR bands of IONP´s, PLA, and PCL are shown in Figure 7a–c and the BM-2 marker clip bands are presented in Figure 7d. The band located at 3340 cm^−1^ comes from O–H end group from PLA and PCL, the bands at 2933 and 2857 cm^−1^ were assigned to asymmetric and symmetric CH_2_ stretching respectively, the band at 1720 cm^−1^ corresponds to a carbonyl stretching (C=O), the located band at 1620 cm^−1^ corresponds to O–H bending vibrations from adsorbed water on the IONs surface, at 1470 and 1364 cm^−1^ were assigned to CH_3_ and CH symmetric and asymmetric bending, the bands at 1292 and 1044 cm^−1^ are related to C–C and C–O stretching from the crystalline and amorphous regions of the copolymer, the band at 1240 cm^−1^ from C–O–C asymmetric stretching, and the band at 1168 cm^−1^ from C–O symmetric stretching and a band at 568 cm^−1^ from the Fe–O surface vibration of the iron oxide core [31,32,33].

**Figure 8:**
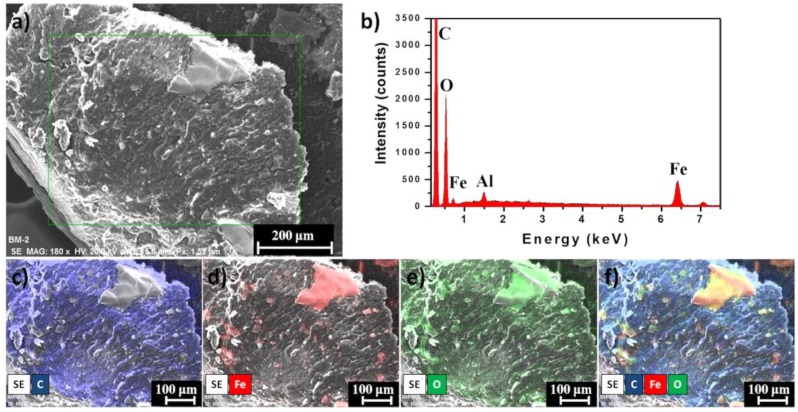
Element mapping of the IONPs on the copolymer: (**a**) SEM image, (**b**) EDS spectrum of complete element distribution, images (**c**–**e**) element maps of C (blue), Fe (red) and O (green), and (**f**) C + Fe + O overlay.

In order to observe the distribution of the IONPs on the copolymer, the elemental distribution of the marker clip prototype was analyzed trough scanning electron microscopy—energy dispersive spectroscopy (SEM-EDS). Figure 8a,b shows the SEM image and EDS spectrum of the marker clip prototype, respectively. The EDS spectrum confirms the existence of carbon (C), iron (Fe), and oxygen (O) signals from the copolymer and IONPs (aluminum signal is from the stub). Figure 8c–e show the EDS mapping of the elemental distribution of C (blue), Fe (red), and O (green), respectively. Mapping images clearly shows that exist a good distribution of the IONPs on the copolymer.

### 3.5. Radiopacity Test of the Marker Clip

**Figure 9:**
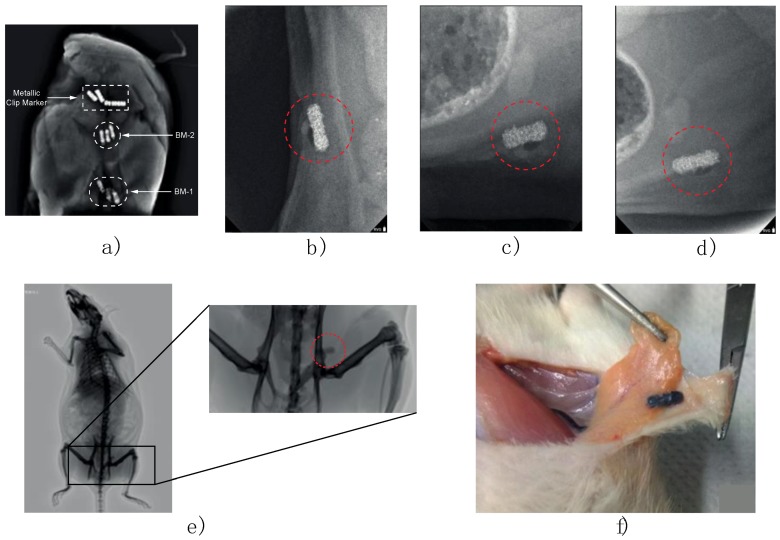
(**a**) Radiopaque properties of composite (BM-1 and BM-2) and commercial marker clips, using a mimetic mammary gland composed of adipose and muscle tissue. X-ray visibility of post-biopsy composite markers after (**b**) one day, (**c**) three months, and (**d**) four months of marker clip insertion, (**e**) Diagnostic mammogram of Wistar rat carrying out the post-biopsy composite maker after insertion. (**f**) Mammary gland dissection at sacrifice, showing marker clip integrity.

**Figure 10:**
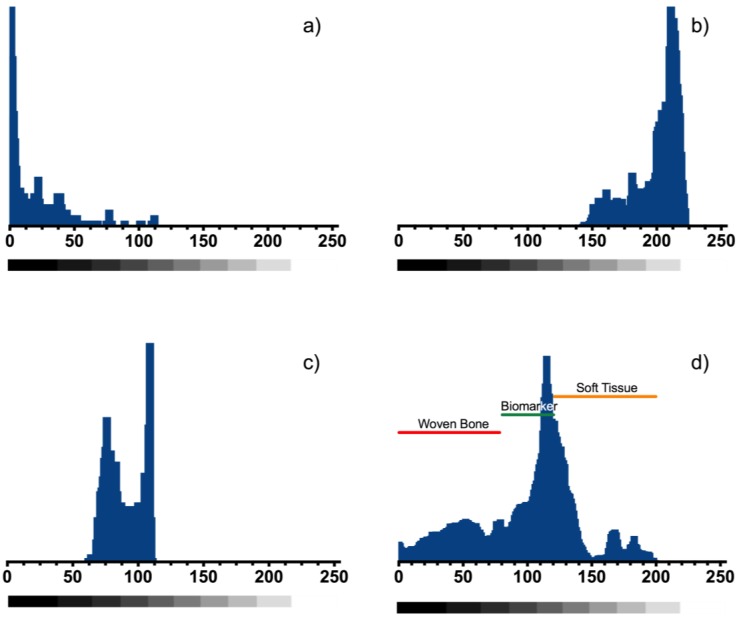
Grayscale values histogram analysis made from zoom image of Figure 9e. (**a**) Woven bone histogram, (**b**) BM-2 marker histogram, (**c**) soft tissue histogram and (**d**) histogram of the complete image and values were the image components (woven bone/BM-2/soft tissue) could be discriminated from each other, these values are presented in Table 3.

The radiopaque properties of the marker clips formulated in this study were evaluated in both in vitro and in vivo phases. For the in vitro phase, two biomarkers (labeled BM-1 and BM-2) and one commercial metallic marker were implanted in a mammary gland model composed by adipose and muscle tissue and can be observed in the X-ray imaging (Figure 9). Also, to verify the in vivo radiopaque capacity of the biomarker, the artifacts were deployed into inguinal mammary gland of female Wistar rats that were maintained under the influence of sex steroids during the experimentation time (4 months). From these last radiopaque images, a grayscale analysis was performed to compare the marker visibility against soft tissue and woven bone as seen in the histograms presented in Figure 10 and Table 3 summarize the relative statistic values from radiopacity contrast.

## 4. Discussion

It is well known, that PLA and PCL are biocompatible and degradable. About the toxicity of the NPs, previous studies have evaluated in vivo and in vitro the cytotoxicity of the IONPs. Mahmoudi et.al reported that in cell culture, concentrations of pristine 40 nm nanoparticles from 500 to 1000 micrograms per milliliter impaired cell proliferation in C6 glioma cells and primary neural cells [34], and in a pre-clinical and safety pharmacokinetics study, they reported that the nanoparticles showed endosomal-lysosomal localization indicating the normal cellular detoxification pathway [35]. In this last report for IONPs toxicity was analyzed at different doses and in different species: rats, dogs, monkeys, and rabbits, showing that iron toxicity was observed only after very high exposure levels. These findings combined with our results on the degradation rate of all samples (PB-1, PB-2, PB-3, and BM-2; see Section 3.2) indicate that nanoparticles release would be very slow, decreasing the possibilities of cellular toxicity within the fat pad of our animal model (the rat), which it could serve as an impediment to nanoparticle migration to the main circulation. Regarding physical characterization; iron nanoparticles (size ~39.6 nm) were found as an advantage for our device. Additionally, XRD patterns indicate that IONPs are mainly conformed by crystalline magnetite (Fe_3_O_4_) as reported before [14], also the superparamagnetic behavior of the synthesized particles with no hysteresis, enhances stability and no migration on the marker giving a possible application as a contrast agent in order to monitor a breast lesion. As mentioned in the introduction section, biomedical applications of composite depend on its ability to degrade in vivo conditions, low toxicity, and appropriate mechanical properties. Degradation of the copolymer is described properly with the model proposed because R-square is >0.99, except for sample PB-3 where a linear model is adequate. PB-2 sample is the most stable polymer mixture (lower weight loss = 7.5%) and it has the highest k. PB-1 polymeric blend has the lowest k value, which indicates that degradation of the copolymer (by hydrolysis) increases with the higher concentration of PLA in the formulation. Finally, PB-3 formulation (lowest amount of PLA) exhibit similar degradation and weight loss compared with PB-1. This could be due that the amount of PLA is not enough to blend with PCL, leading to a greater degradation and weight loss during the time of experimentation. Our findings in degradation rate suggest that the composite formulated with 95% PCL and 5% PLA (PB-2) has the proper properties for application as a biopsy marker, because after 126 days of experimentation only presented the lowest percentage of weight loss. On the other hand, when formulation PB-2 is filled with 36.86 mg of IONPs, the weight loss is reduced from 7.5% to 3.1%. These results suggest that the incorporation of IONPs into the composite formed a novel hybrid material with different physicochemical properties than the original formulation (PB-2). These changes had been reported in other studies with different purposes; the incorporation of metallic nanoparticles into polymers can retard or accelerate thermal decomposition of materials, as reported by Lee et al [19] with Pd nanoparticles.

From mechanical properties, the sample with less PCL concentration (PB-1) has more flexibility, higher strain proportionality limit, and low compressive strength. The sample with the lowest PLA content (PB-3) became stiffer, decreasing the strain proportionality limit value, but increasing its compressive strength. On the other hand, formulation PB-2 showed the highest stress and strain proportionality limit, and showed the highest value for maximum compression strength; then, this formulation has the most adequate mechanical properties for the purpose of this work and in consequence, was chosen for in vivo analyses.

One of the most remarkable findings obtained from thermograms is that the composite marker is thermally stable at temperatures below 100 °C, which may suggest that our PLA/PCL composite it could be stable during experimentation at corporal temperatures (~37 °C). As mentioned above, composite marker showed DSC profiles with series of thermal transitions different from pure polymers profiles; it can be observed a peak shifted from 55 to 45 °C, that could be related to the moisture evaporation presented in PCL, also an endothermic peak at 145 °C, which could be related to the melting point of PLA, and finally the peaks related to structural decomposition of both pure polymers that exhibited lower intensity. This may suggest copolymer has a lower thermal decomposition that PLA and PCL. In other words, the results suggest that the structural organization of copolymer formulation is more stable to thermal degradation which is probed from the FTIR spectra of the marker where it can be seen the characteristic bands of PLC overlapping PLA, due to the composition of the marker and similarities on functional groups but also the integration of the IONPs can be seen as an additional band on the spectra. From the EDS analysis of the IONPs on the surface of the marker clip, they are uniformly distributed on the copolymer and not agglomeration was observed.

As it can be observed in the X-ray imaging (Figure 9a), only the composite marker BM-2 exhibited a similar image contrast and sharpness in comparison to a commercial metallic marker. This could be achieved because IONPs concentration added to composite was higher for BM-2 than BM-1. It is important to mention that the concentrations of IONPs, used to formulate the composite biopsy markers of this study, were always below to the half-lethal concentration of oxide iron nanoparticles reported by other authors [20,23]. Based on these findings, it was decided to use the BM-2 composite marker to evaluate its marker properties in the in vivo model. In addition, about the inflammation; in Figure 9f it is not observed any macroscopic sign of foreign body inflammation, edema formation or infection within the fat pads or surrounding areas, neither darkened body areas were observed. After removal of the clips, no macroscopic signs of necrosis were seen and none experiment-related death was recorded. Also, as it can be observed in Figure 9, the composite markers development in this study were readily visible in mammograms, showing good visibility and contrast properties during the 4 months of in vivo implementation as post-biopsy markers. An important finding is that composite marker clips were not displaced in the 4 months of in vivo experiment; there is an important parameter in optimal patient care reducing side effects in breast tissue. These results indicate that the composite post-biopsy marker formulated in this study could be an alternative material option for marker clips.

## 5. Conclusions

In this study, a formulation for a novel material for marker clips was proposed. Using IONPs, it was possible to achieve the radiopacity property of the marker clip. However, we recommend using nanoparticles with sizes around 80 nm for medical applications. PLA was developed through polymerization that was confirmed by thermal analysis. Our results showed that BM-2 sample (95% PCL/5% PLA, w/w) had the most adequate thermal, mechanical and degradability properties. IONPs into PLA/PCL copolymer to enhance the stability of the formulation, this finding can be used for long-term life expectancy studies of marker clips. In vitro, radiopaque experiments showed that BM-2 composite had the best performance. In vivo experiments showed that, after five months, the marker clip prototype maintained its shape, visibility and contrast properties. Also, it did not migrate. In consequence, a novel formulation of composite (PLA/PCL/IONPs) is suitable for further studies as an alternative material for marker clips for breast cancer lesions.

## Figures and Tables

**Table 1:** Results of degradation rate against hydrolysis rates and percentage. *Total weight loss percentage after the elapsed 120 days of the experiment.

**Exponential Fit**			
Sample	*k* (24 h^−1^)	*R* ^2^	Weight loss (%)*
PB-1	0.0379	0.9903	13.5 ± 0.87
PB-2	0.1273	0.9930	7.5 ± 0.91
BM-2	0.1095	0.9920	3.1 ± 0.82
**Linear Fit**			
Sample	m (24 h^−1^)	*R* ^2^	Weight loss (%)
PB-3	−0.001054	0.9923	13.8 ± 1.06

Results show the mean value ± SE from three samples.

**Table 2:** Average values obtained in the mechanical compression tests on cylindrical specimens ^1^.

Sample	Height (mm) [±0.02]	Diameter (mm) [±0.01]	Area (mm^2^) [±0.3]	E ^2^ (MPa)	YS ^3^ (MPa)	CS ^4^ (MPa)	SA ^5^ (%)
PB-1	3.97	19.62	302.33	1.361 ± 0.07	0.0581 ± 0.010	0.112 ± 0.02	4.18
PB-2	3.59	19.91	311.34	1.480 ± 0.06	0.0622 ± 0.008	0.286 ± 0.02	5.30
PB-3	3.92	19.90	311.03	1.761 ± 0.07	0.0581 ± 0.009	0.169 ± 0.02	3.36
BM-2	3.82	19.80	307.91	1.574 ± 0.09	0.0602 ± 0.011	0.321 ± 0.03	4.45

^1^ Results show the mean value ± SE from three samples; ^2^ Young’s modulus; ^3^ Yield Stress; ^4^ Compressive Stress; ^5^ Strain.

**Table 3:** Grayscale bit values from the analysis of radiopaque images.

Sample	Minimun	Maximun	Average	Relative Radiopacity Against Woven Bone (%)
Soft tissue	139	222	181	19.88
BM-2	63	118	119	30.25
Woven Bone	0	72	36	100
